# Promotion of Bone Defect Repair Using Decellularized Antler Cancellous Bone Loaded with Deer Osteoglycin

**DOI:** 10.3390/biom15081124

**Published:** 2025-08-04

**Authors:** Yusu Wang, Ying Zong, Weijia Chen, Naichao Diao, Quanmin Zhao, Boyin Jia, Miao Zhang, Jianming Li, Yan Zhao, Zhongmei He, Rui Du

**Affiliations:** 1 College of Chinese Medicinal Materials, Jilin Agricultural University, Changchun 130118, China; wangyusu@mails.jlau.edu.cn (Y.W.); zongying@jlau.edu.cn (Y.Z.); weijiac@jlau.edu.cn (W.C.); diaonaichao@jlau.edu.cn (N.D.); zhaoquanmin@jlau.edu.cn (Q.Z.); zhangmiao96@mails.jlau.edu.cn (M.Z.); lijianming773@jlau.edu.cn (J.L.); zhaoyan@jlau.edu.cn (Y.Z.); 2 College of Animal Science and Technology, Jilin Agricultural University, Changchun 130118, China; jiaboyin@jlau.edu.cn; 3 College of Pharmacy, Yanbian University, 997 Park Road, Yanji 133002, China

**Keywords:** deer osteoglycin, bone defect repair, bioactive factors, decellularized antler cancellous bone, enhanced bone healing

## Abstract

The combination of scaffold materials and bioactive factors is a promising strategy for promoting bone defect repair in tissue engineering. Previous studies have shown that osteoglycin (OGN) is highly expressed in the bone repair process using deer antler as an animal model of bone defects. It suggests that OGN may be a key active component involved in the bone repair process. The aim of this study was to investigate whether deer OGN (dOGN) could effectively promote bone regeneration. We successfully expressed dOGN using the *E. coli* pET30a system and evaluated its biological activity through cell proliferation and migration assays. At a concentration of 5 μg/mL, dOGN significantly promoted cell proliferation and migration. We then incorporated dOGN onto decellularized antler cancellous bone (DACB) scaffolds and assessed their osteogenic potential both in vitro and in vivo. The results indicated that dOGN loading enhanced cell proliferation, adhesion, and osteogenic activity. In vivo experiments confirmed that the dOGN-DACB scaffold significantly improved bone regeneration compared to DACB alone. This study demonstrates that dOGN-loaded DACB scaffolds hold great potential for clinical applications in treating critical-sized bone defects by mimicking the rapid regenerative properties of deer antlers.

## 1. Introduction

Repair of bone defects represents a major clinical challenge in orthopedics, particularly in cases involving trauma, infection, and tumor resection [1,2]. These situations often lead to large bone defects that are refractory to self-healing. Currently, autologous bone grafting remains the ‘gold standard’ for the clinical treatment of bone defects [3]. It has the advantages of sharing the same spatial structure as the host bone tissue and containing essential bioactive components for promoting bone regeneration [4,5]. Nevertheless, the limitations of autologous bone grafting, such as a limited donor area source and donor-area complications, have restricted its extensive application in clinical practice [6].

To overcome these limitations, xenogeneic cancellous bone materials with a bone structure similar to that of autologous bone tissue have been developed as an alternative [7,8,9]. The xenogeneic cancellous bone material, after decellularization, retains the three-dimensional (3D) structure and extracellular matrix components of native bone tissue, providing an optimal environment for osteoblast attachment, proliferation, and differentiation [10,11]. Our preliminary study has found that decellularized antler cancellous bone (DACB) may be a more ideal scaffold material for bone grafting [12]. It is well known that antlers are bony organs that undergo cyclical regeneration each year on the skulls of deer, and the growth rate of their bone tissue is among the fastest observed in medium- to large-sized mammals [13]. The rapid regeneration of antlers is primarily driven by the rich vascular network within the antler [14]. During the calcification of antlers in autumn, the internal blood vessels progressively regress, ultimately leaving only the cancellous bone structure [15]. This cancellous bone exhibits a porous architecture and provides a microenvironment favorable for angiogenesis [16]. Vascularization plays a crucial role in bone regeneration, as the newly formed vascular network not only supplies oxygen and nutrients to the skeletal system and removes metabolic waste but also transports hormones, growth factors, and neurotransmitters, thereby supporting cell survival and enhancing their functionality [17].

In an effort to further boost the repair efficiency of bone tissue, bone graft materials are frequently combined with bioactive factors to replicate the microenvironment inherent in autologous bone grafts. Thus, exploring new osteogenic induction factors post-bone-grafting is one of the key challenges in bone repair [18]. The abundant vascularity and rapidly regenerating bone tissue in deer antler tissues offer a novel research avenue for addressing this problem. In a previous study, we utilized growing antlers as a bone defect model. Through transcriptomic sequencing analysis, we identified that the expression of osteoglycin (OGN) was significantly upregulated during antler regenerative repair [12]. Previous research has indicated that OGN is an extracellular matrix protein belonging to the small leucine-rich repeat protein family (SLRP) [19]. It plays a pivotal role in bone tissue development and regeneration [20]. However, there have been relatively few studies on the application of OGN in bone tissue engineering, and its mechanism of action remains unclear.

The aim of this study was to investigate the potential of DACB materials loaded with dOGN in bone repair. We used a prokaryotic protein expression system to obtain dOGN and loaded it into DACB, and systematically evaluated the physicochemical properties, biocompatibility, osteogenic activity, and bone repair effect of dOGN-DACB composites. The innovative combination of dOGN and DACB in this study aims to investigate the promotional role of dOGN as an osteoinductive factor in bone repair.

## 2. Materials and Methods

### 2.1. Materials

Decellularized antler cancellous bone matrix (DACB) has been prepared in our previous study. Human-osteoglycin (hOGN) was purchased from NovoPro Biotechnology Co., Ltd. (Shanghai, China). Collagen Type I (Col I) Polyclonal antibody and secondary antibodies were purchased from Proteintech Biotechnology Co., Ltd. (Wuhan, China). Rabbit anti-osteocalcin (OCN) antibody, rabbit anti-osteopontin (OPN) antibody, and FITC-Goat Anti-Rabbit IgG (H + L) were purchased from FineTest Biotechnology Co., Ltd. (Wuhan, China).

### 2.2. Purification of dOGN

The gene encoding dOGN was inserted into Pet30a and transformed into *E. coli* BL21. After overnight amplification of *E. coli* BL21, protein expression was induced with 0.1 mM isopropyl β-d-thiogalactopyranoside (IPTG, Solarbio, Beijing, China) at 37 °C for 4 h. The precipitate from lysed *E. coli* BL21 was collected and purified by nickel chelate chromatography using a 6 × His tag. The dOGN protein was eluted with gradient imidazole and the effluent was collected. Subsequently, the collected proteins were dialyzed overnight for refolding. The purity and molecular weight of recombinant proteins were analyzed by 12% SDS-PAGE.

### 2.3. Bioactivity Assay of dOGN In Vitro

#### 2.3.1. Cell Proliferation and Toxicity Assay

C3H10T1/2 cells (2 × 10^3^ cells/well) were seeded in 96-well plates and cultured in complete or dOGN-containing medium (1, 3, 5 μg/mL) for 1, 3, 5, 7 days. Cell viability was assessed using CCK-8 kit (Biosharp, Hefei, China). At each time-point, CCK-8 reagent was added to each well and incubated at 37 °C for 1 h before measuring absorbance at 450 nm. On day 3, cells were stained with Calcein/PI Cell Viability/Cytotoxicity Assay Kit (Beyotime, Shanghai, China). Cell counting was performed using ImageJ (version 1.51j, NIH, Bethesda, MD, USA).

#### 2.3.2. Cell Migration

C3H10T1/2 cells were seeded in 6-well plates at 2 × 10^5^ cells/well and cultured to confluence. A scratch was made on the monolayer with a P200 pipette tip and washed thrice with PBS to remove detached cells. Medium with different dOGN concentrations and control medium were added. Digital images of the cells were taken at 0 h and 24 h post-wounding using microscope (Leica, DMi8, Wetzlar, Germany). They were then analyzed using ImageJ software (version 1.51j, NIH, Bethesda, Md, USA). The migration area (%) was calculated according to the formula: migration area = (M1 − M0)/M0 × 100%, where M1 represents the wound area at 24 h and M0 represents the wound area at 0 h.

Furthermore, cell migration was assayed using 24-well Transwell plates (Corning, NY, USA). Around 1 × 10^5^ cells in 200 μL of serum-free medium were seeded in the upper chamber, while 500 μL of medium with 10% FBS and various dOGN concentrations was added to the lower chamber. After 24 h incubation, cells on the upper layer were removed with a cotton swab. Cells beneath the membrane were fixed with 4% paraformaldehyde for 20 min and stained with 0.1% crystal violet for 15 min. Migrated cells were counted using ImageJ software after photographing under a microscope (Leica, DMi8, Wetzlar, Germany).

### 2.4. OGN Loading and Characterization on DACB

DACB was obtained from completely ossified deer antlers. Detailed procedure for preparing DACB has been described in our previous study [12]. In brief, antler cancellous bone was subjected to a freeze-thaw cycle, followed by sequential washing with 5% hydrogen peroxide, 4% SDS, and 1% Triton X-100. Subsequently, the samples were incubated in a nucleic acid removal solution containing 50 U/mL DNase I (Macklin, Shanghai, China) and 1 U/mL RNase (Macklin, Shanghai, China). Finally, the samples were lyophilized and sterilized using 25 kGy cobalt-60 gamma radiation (Gammacell 220, MDS-Nordion, Ottawa, ON, Canada).

With hOGN serving as the control bioactive factor, hOGN and dOGN were individually loaded onto DACB. Briefly, 0.5 g of DACB was immersed in both hOGN and dOGN at a concentration of 5 μg/mL for 12 h. The obtained composites were then freeze-dried using a lyophilizer. Scanning electron microscopy (SEM, SU−70, Tokyo, Japan) was used to observe the microstructure of the samples and the distribution of bioactive factors on the DACB scaffolds. SEM images were acquired at an accelerating voltage of 10 kV. Fourier transform infrared spectroscopy (FTIR; Avatar 360, Nicolet, WI, USA) was employed to characterize the infrared absorption spectra of dOGN and hOGN adsorbed on DACB. The measurements were conducted with 32 scans, a spectral range of 4000–400 cm^−1^ and a resolution of 4 cm^−1^.

### 2.5. In Vitro Release Assay

The composites were immersed in 2 mL of PBS solution (pH 7.4) and gently shaken at 100 rpm at 37 °C. Samples were retrieved at predetermined time points: 0.5 h, 1 h, 2 h, 3 h, 6 h, and 12 h, and on days 1, 2, 4, and 7. At each sampling point, 1 mL of the release medium was carefully collected, and an equivalent volume of fresh PBS solution was added to maintain consistent experimental conditions. As reported in previous studies, the amount of OGN released was quantified using a Human OGN Immunoassay Kit (Youxuan, Shanghai, China) and measured with a BioTek Synergy HTX microplate reader (BioTek, Winooski, VT, USA) [21].

### 2.6. Attachment and Proliferation of Cells

The DACB composites with attached OGN were cut into pieces measuring 6 mm × 4 mm and placed into a 24-well plate. C3H10T1/2 suspension (2 × 10^4^ cells/well) was seeded onto experimental scaffolds. After 3 days, the morphology of cells attached to the samples was observed by SEM. Proliferation assays were performed in 96-well plates at a seeding density of 2 × 10^3^ cells/well. On days 1, 3, 5, and 7 after seeding, cell proliferation activity was measured using the CCK-8 kit, and the absorbance values of each group were measured at 450 nm using the multifunctional microplate scanner.

### 2.7. Cytotoxicity Assessment

C3H10T1/2 cells were seeded onto the surface of dOGN-DACB and hOGN-DACB at a density of 2 × 10^4^ cells and cultured in DMEM complete medium for 3 days. The cells were then stained according to the instructions of the Live/Dead Staining Kit (Beyotime, Shanghai, China). Cell morphology was observed under a fluorescence microscope (Leica, DMi8, Wetzlar, Germany). The number of live and dead cells was quantified using ImageJ software (version 1.51j, NIH, Bethesda, MD, USA).

### 2.8. Osteogenic Differentiation In Vitro

To evaluate the bioactivity of the released bioactive factors, an indirect co-culture method was used to preliminarily assess the osteogenic effect. DACB scaffolds with hOGN or dOGN were immersed in osteogenic induction medium, with DACB as the control. After 2 days, the conditioned medium containing the eluted bioactive factors was collected to treat preseeded C3H10T1/2 cells (*n* = 3, 2 × 10^4^ cells/well) in 24-well plates. On day 7, Alkaline Phosphatase (ALP) activity was measured using an ALP assay and staining kit (Beyotime, Shanghai, China). On day 14, cells were fixed with 4% paraformaldehyde for 15 min, stained with 1% Alizarin Red S (ARS, Solarbio, Beijing, China) at room temperature for 30 min, washed with PBS and imaged. The mineralized nodules were dissolved using a 10% solution of hexadecylpyridinium chloride monohydrate (Yuanye, Shanghai, China) and the absorbance at 570 nm was measured to quantify the mineralization.

On day 14, the conditioned medium was removed from the 24-well plates. Cells were fixed with 4% paraformaldehyde for 15 min, blocked with 1% BSA for 1 h, and permeabilized with 0.1% Triton X-100 for 15 min. To detect Col I, OPN and OCN, cells were incubated with primary antibodies at 4 °C overnight, followed by incubation with secondary antibodies for 1 h after removing the primary antibodies. Nuclei were stained with DAPI for 10 min. Finally, the samples were observed and imaged using a fluorescence microscope (Leica DMi8, Wetzlar, Germany).

For further investigating the osteogenic effect, direct co-culture method was performed. Cells were inoculated on the surface of the composite material after 7 and 14 days of culture, ALP activity was measured using an ALP assay and staining kit. The composite scaffold materials stained with ALP were observed and photographed using a cell phone (HONOR 30, Honor, Shenzhen, China).

RT-PCR was performed to measure the expression levels of osteogenesis-related mRNAs and further evaluate the effects of the composites on osteogenic differentiation. After 7 and 14 days of culture, total RNA was extracted from the cells using TRIzol reagent (Cwbio, Beijing, China) and subsequently reverse-transcribed into cDNA. The mRNA expression levels of Col I, OPN, and OCN were quantified using a fluorescence-based quantitative PCR kit (Takara, Dalian, China). Relative mRNA expression levels were normalized using the 2^−ΔΔCt^ method, with glyceraldehyde-3-phosphate dehydrogenase (GAPDH) as the housekeeping gene. The primer sequences are listed in Table S1.

### 2.9. In Vivo Osteogenic Assessment

In this study, all animal procedures were approved by the Animal Research Committee of Jilin Agricultural University (AEC 2021101103). Twenty-four male Sprague-Dawley (SD) rats weighing 250 ± 30 g were randomly divided into four groups: Control, DACB, hOGN-DACB, and dOGN-DACB. All rats were anesthetized by intraperitoneal injection of 1% pentobarbital (50 mg/kg). A 2 cm longitudinal incision was made along the lateral aspect of the distal femur to expose the lateral femoral condyle. Using a drill bit, a bone defect measuring 3 mm in width and 4 mm in depth was created at the incision site. DACB, dOGN-DACB, and hOGN-DACB scaffolds (sized to match the defect) were implanted into the defect sites, while the control group received no scaffold implantation. Rats were euthanized with an overdose of sodium pentobarbital at 4 and 8 weeks post-surgery. Subsequently, the femoral condyle specimens were subjected to a micro-CT scanner (SkyScan 1176, Bruker, Kontich, Belgium) with the following parameters: an X-ray voltage of 80 kV, a current of 100 mA, an exposure time of 200 ms, and a scan resolution of 10 μm. Following micro-CT examination, the samples from each group were placed in an EDTA solution for decalcification for 10 days. The decalcified samples were embedded in paraffin and sectioned into 5-μm-thick slices. The newly formed trabecular structure was observed via H&E and Masson staining. For immunohistochemical (IHC) staining, antibodies against CD31, Col I, OPN, and OCN were used according to the manufacturer’s instructions. Biosafety was evaluated by performing H&E staining on five important organs.

### 2.10. Statistical Analysis

All experimental data were statistically analyzed using GraphPad Prism 9.5.1 software. Data are presented as the mean ± standard deviation (SD). One-way or two-way analysis of variance (ANOVA) was performed to determine statistically significant differences.

## 3. Results and Discussion

In bone tissue engineering, scaffold materials and bioactive molecules play a crucial role in promoting bone repair. The development of decellularized cancellous bone provides an ideal bone graft scaffold for biomaterials in bone regeneration. DACB preserves the biochemical microenvironment and biophysical properties of antler during its rapid regeneration, making it a promising candidate for the development of novel bone graft materials. In this study, DACB was functionalized by loading its surface with the bioactive molecule OGN to enhance its synergistic effects. This modification aimed to improve cell adhesion, proliferation, and osteogenic differentiation, thereby optimizing the scaffold’s potential for bone tissue regeneration.

### 3.1. Preparation and Bioactivity Assays of dOGN In Vitro

**Figure 1:**
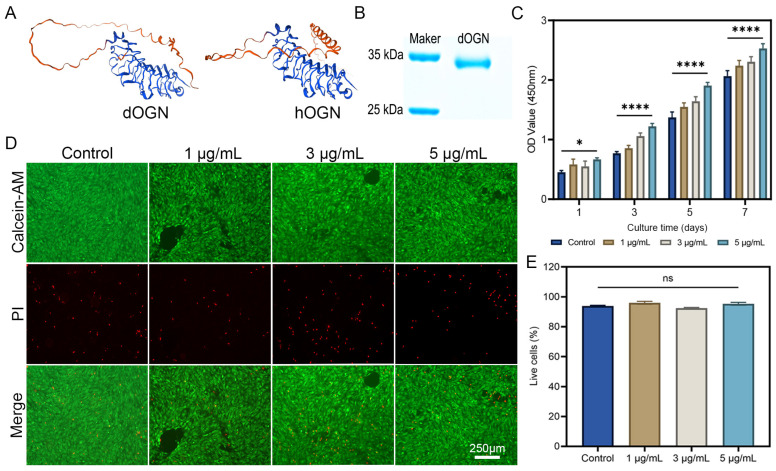
Preparation and properties of the dOGN. (**A**) Homologous modeling for predicting the 3D structure of proteins. (**B**) The molecular weight of dOGN examined by SDS-PAGE. (**C**) CCK-8 assay was performed to assess the proliferation of C3H10T1/2 cells co-cultured with different concentrations of dOGN for 1, 3, 5, and 7 days (*n* = 3). (**D**) Live/dead (green/red) fluorescence staining images of C3H10T1/2 cells cultured in different concentrations of dOGN for 3 days. scale barn: 250 μm. (**E**) Quantitative analysis of the live/dead cell staining assay. ns, not significant, * *p* < 0.05 and **** *p* < 0.0001.

The 3D structural model of dOGN protein was predicted using WeMol. The results demonstrated that the 3D structure of dOGN closely resembled the spatial structure of hOGN (Figure 1A). BLAST comparative analysis (BLAST—2.16.0, National Center for Biotechnology Information, Bethesda, USA) showed that the two shared 91% sequence similarity. The recombinant dOGN protein was successfully obtained using an *E. coli* expression system. The predicted molecular weight of dOGN was 33.26 kDa, and SDS-PAGE analysis confirmed that the purified dOGN had an approximate molecular weight of 34 kDa (Figure 1B), consistent with previously published literature [22]. The biological activity of dOGN was then evaluated in vitro. As shown in Figure 1C, dOGN exhibited strong bioactivity in promoting cell proliferation, with a significant proliferation-enhancing effect observed at an dOGN concentration of 5 μg/mL. Subsequently, the cytotoxicity of different dOGN concentrations was assessed. The results indicated that dOGN did not exhibit significant cytotoxic effects on the cells (Figure 1D). Quantitative analysis of fluorescence images also showed no significant differences between the experimental groups (Figure 1E). 

**Figure 2:**
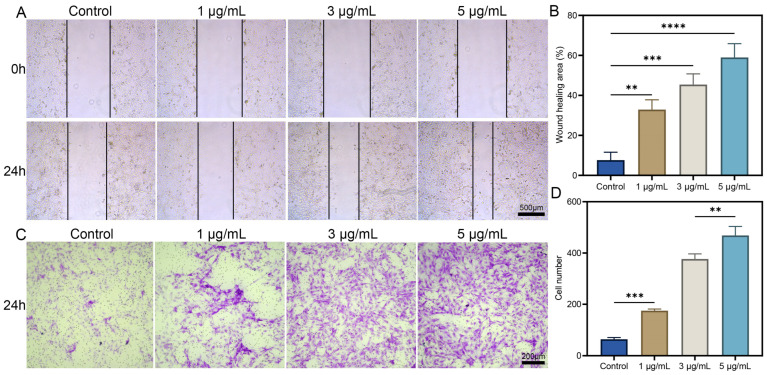
In vitro cellular effects of different concentrations of dOGN. (**A**) Representative micrographs of wound edges in scratch assays for each group. Scale bar: 500 μm. (**B**) Quantification of wound closure rate. (**C**,**D**) Representative images and corresponding quantitative analysis of Transwell migration assays in C3H10T1/2 cells (*n* = 3). Scale bar: 200 μm. ** *p* < 0.01, *** *p* < 0.001 and **** *p* < 0.0001.

The directional migration of mesenchymal stem cells (MSCs) plays a crucial role in bone repair [23]. To assess the effect of dOGN on the migratory capacity of C3H10T1/2 cells, scratch and Transwell assays were performed. Cells were co-cultured with different concentrations of dOGN for 24 h. Compared with the control group, no significant increase in cell migration was observed at an dOGN concentration of 1 μg/mL. However, at concentrations of 3 and 5 μg/mL, a significant enhancement in cell migration was detected (Figure 2A), with the most pronounced effect observed at 5 μg/mL (Figure 2B). These findings indicate that dOGN at a concentration of 5 μg/mL exhibits optimal bioactivity in promoting both cell proliferation and migration, as further supported by the Transwell assay results (Figure 2C,D). Therefore, dOGN was loaded onto DACB at a concentration of 5 μg/mL for subsequent studies. Previous studies have also shown that OGN concentrations ranging from 3 to 6 μg/mL significantly promote cell proliferation [24].

### 3.2. Loading Characteristics of Bioactive Factors onto DACB

**Figure 3:**
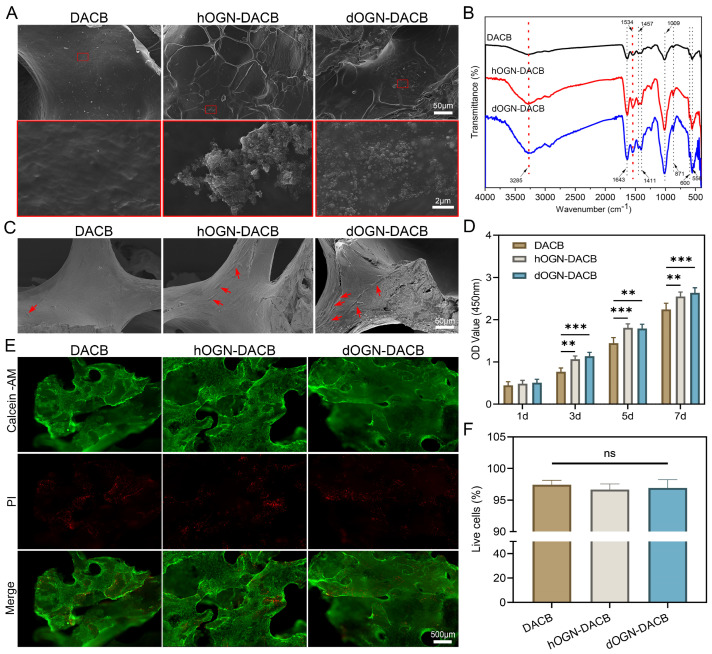
In vitro bioactivity of C3H10T1/2 cells on DACB scaffolds loaded with bioactive factors. (**A**) SEM images of the DACB surface before and after loading with bioactive factors. Scale bar: 50 μm. Enlarged views of selected areas are shown in red boxes (Scale bar: 2 μm). (**B**) FT-IR spectra of DACBs loaded with different bioactive factors. (**C**) SEM images of C3H10T1/2 cells after 3 days of culture on different scaffolds. Scale bar: 50 μm. Red arrows indicate attached cells. (**D**) Proliferation of C3H10T1/2 cells on different scaffolds evaluated using the CCK-8 assay. (**E**) Live/dead staining images of C3H10T1/2 cells cultured on various scaffolds for 3 days. Scale bar: 500 μm. (**F**) Quantitative analysis of live/dead staining results. ns, not significant, ** *p* < 0.01 and *** *p* < 0.001.

The dOGN and hOGN at a concentration of 5 μg/mL were selected for loading onto DACB scaffold material to prepare composite scaffolds. SEM and FTIR analyses were performed on DACB before and after loading to assess the incorporation of OGN onto the scaffold. SEM images showed that the surface of DACB loaded with OGN bioactive factors exhibited small OGN particles distributed throughout the scaffold material, whereas the unmodified DACB surface appeared smoother (Figure 3A). To further confirm the incorporation of OGN, FTIR analysis was conducted on DACB composites loaded with dOGN and hOGN, using unloaded DACB as a control. The FTIR spectra of the loaded DACB scaffolds indicated characteristic peaks corresponding to OGN, confirming successful incorporation (Figure 3B). The results showed an amide A-band at 3285 cm^−1^, attributed to the N-H vibration of acetamide [25]. This peak is commonly observed in proteins and indicates the presence of peptide bonds in the OGN bioactive factors loaded onto the DACB scaffold. Additionally, peaks at 1643 cm^−1^ and 1535 cm^−1^ indicated the presence of amide I (-C=O) and amide II (-N-H) groups, respectively [26]. Furthermore, 566 and 600 cm^−1^ were the bending vibration absorption peaks of PO_4_^3−^ in hydroxyapatite, 871 cm^−1^ was the bending vibration absorption peak of CO_3_^2−^, 1009 cm^−1^ was the symmetrical stretching absorption peak of PO_4_^3−^, and 1411 and 1457 cm^−1^ were the stretching vibration absorption peaks of CO_3_^2−^ [27,28,29]. Moreover, FTIR analysis results showed that the peak intensity of the hOGN-DACB and dOGN-DACB were higher than that of DACB, indicating the successful loading of growth factors onto the material. In addition, both hOGN-DACB and dOGN-DACB demonstrated sustained release profiles (Figure S1). Biologically active factors can typically be non-covalently incorporated into scaffolds through three methods: physical embedding, physical adsorption, and ionic complexation [30]. Among these, direct physical adsorption of biologically active factors onto decellularized cancellous bone is the most clinically feasible, effective, and commonly used approach for bone defect repair [31].

### 3.3. Cytocompatibility Test

In vitro biocompatibility was assessed by incubating C3H10T1/2 cells on DACB, hOGN-DACB, and dOGN-DACB surfaces. As shown in Figure 3C, SEM images after 3 days of incubation showed that the number of cells attached to the original DACB surface was low. In contrast, hOGN-DACB and dOGN-DACB had a greater number of cells on them, exhibited enhanced adhesion, and presented an expanded morphology with more pseudopods. Subsequently, the proliferation-promoting effect of OGN-loaded composites on cells was further confirmed by CCK-8 assay (Figure 3D). The results of live/dead staining and quantitative analysis indicated that the composites had good biocompatibility (Figure 3E,F).

### 3.4. Osteogenic Differentiation Effect In Vitro

Ideal bone implants not only need to possess good cytocompatibility and biocompatibility, but also demonstrate excellent osteogenic activity [32]. With this in mind, we evaluated the ability of composite scaffolds loaded with the bioactive factor dOGN to promote osteogenic differentiation in an in vitro setting, with the aim of gaining insight into their potential for bone regeneration applications.

**Figure 4:**
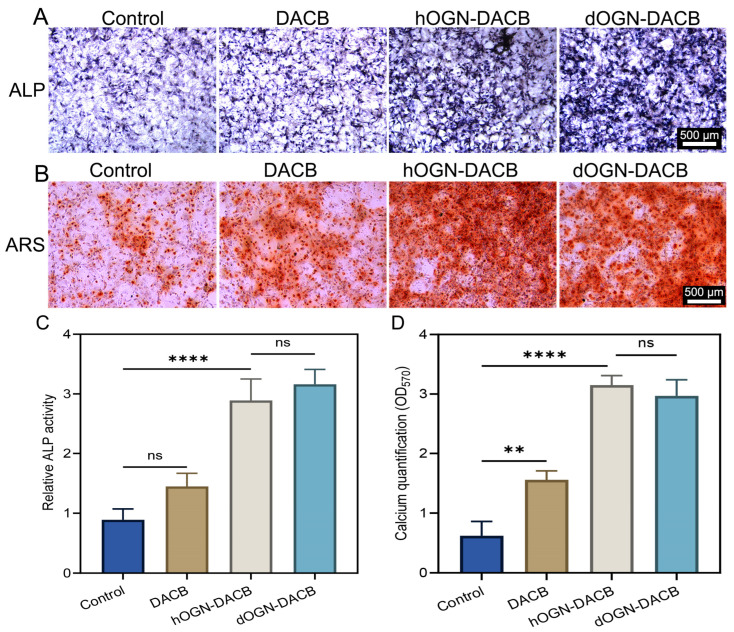
Osteogenic differentiation effect assessment via indirect co-culture method in vitro. (**A**) Representative images of ALP staining of C3H10T1/2 culture with different osteogenic conditioned media for 7 days. (**B**) Representative images of ARS staining of C3H10T1/2 culture with different osteogenic conditioned media for 14 days. (**C**) Quantitative analysis of the ALP activity. (*n* = 3). (**D**) Quantitative analysis of calcium deposition by ARS staining (*n* = 3). ns, not significant, ** *p* < 0.01, and **** *p* < 0.0001.

In our previous study, we demonstrated that DACB promotes osteogenic differentiation of cells [12]. In the present study, the composite material prepared by loading dOGN onto the surface of DACB further enhanced ALP activity significantly. Quantitative analysis of ALP activity revealed results that were highly consistent with the corresponding staining images (Figure 4A,C), clearly indicating that dOGN-DACB had a more pronounced effect in promoting early osteoblast differentiation. After 14 days of co-culture, ARS staining results showed a similar trend to the ALP activity analysis, with a marked increase in the formation of red calcium nodules in the dOGN-DACB and hOGN-DACB groups (Figure 4B). Quantitative analysis further confirmed that mineralization of C3H10T1/2 cells was significantly enhanced in the composite groups (Figure 4D).

**Figure 5:**
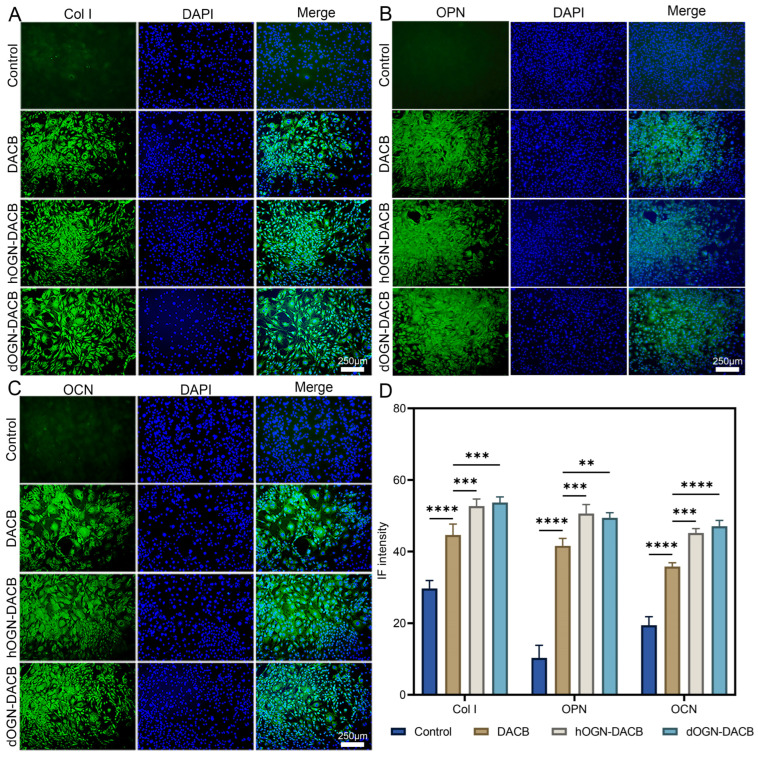
In vitro osteogenic activity of composite scaffolds. (**A**–**C**) Representative immunofluorescence staining images of Col I, OCN, and OPN proteins after 14 days of culture. Nuclei and target proteins are labeled with blue and green fluorescence, respectively. (**D**) Quantitative positive antibody expression for the corresponding proteins. Scale bar: 250 μm. ** *p* < 0.01, *** *p* < 0.001, and **** *p* < 0.0001.

In addition, the expression of intracellular osteogenic proteins was assessed by immunofluorescent labeling of Col I, OPN, and OCN [33,34]. As shown in Figure 5A, the expression levels of Col I in the dOGN-DACB group were significantly higher than those in the control and DACB groups. The expression of OPN and OCN also showed the same trend (Figure 5B,C). Overall, these results clearly indicate that both hOGN-DACB and dOGN-DACB possess stronger osteogenic differentiation-promoting capabilities compared to the control and DACB groups (Figure 5D).

**Figure 6:**
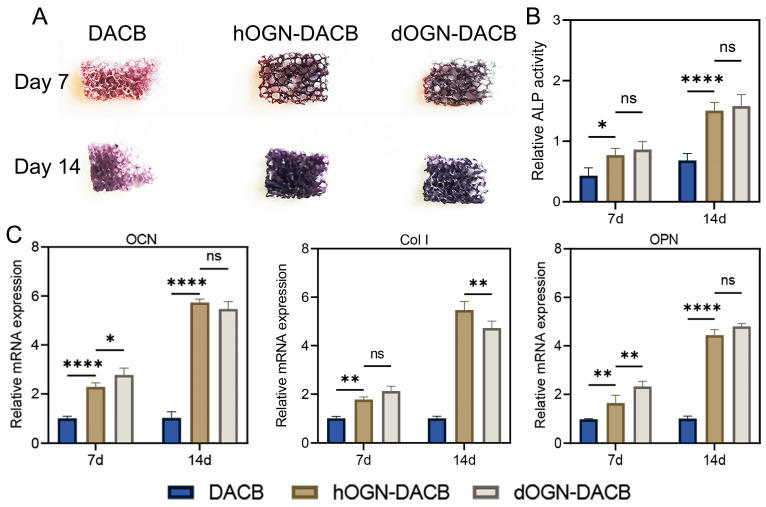
Osteogenic differentiation effect assessment via direct co-culture method in vitro. (**A**) BCIP/NBT staining images of C3H10T1/2 culture on different scaffolds for 7 and 14 days. (**B**) Quantitative analysis of the ALP activity. (**C**) Relative mRNA expression of OCN, Col I, and OPN in C3H10T1/2 culture on different scaffolds for 7 and 14 days. ns, not significant, * *p* < 0.05, ** *p* < 0.01, and **** *p* < 0.0001.

To further evaluate the in vitro osteogenic potential, C3H10T1/2 was inoculated onto different scaffolds and subjected to ALP activity analysis and RT-qPCR analysis. The results showed that the ALP activity analysis results showed the same trend as the above results (Figure 6A,B). Most importantly, RT-qPCR results showed that osteogenic genes (including Col I, OPN, and OCN) were significantly upregulated in the dOGN-DACB group (Figure 6C). Overall, in vitro studies indicate that dOGN and hOGN have similar osteogenic differentiation capabilities.

### 3.5. Bone Healing Effect In Vivo

**Figure 7:**
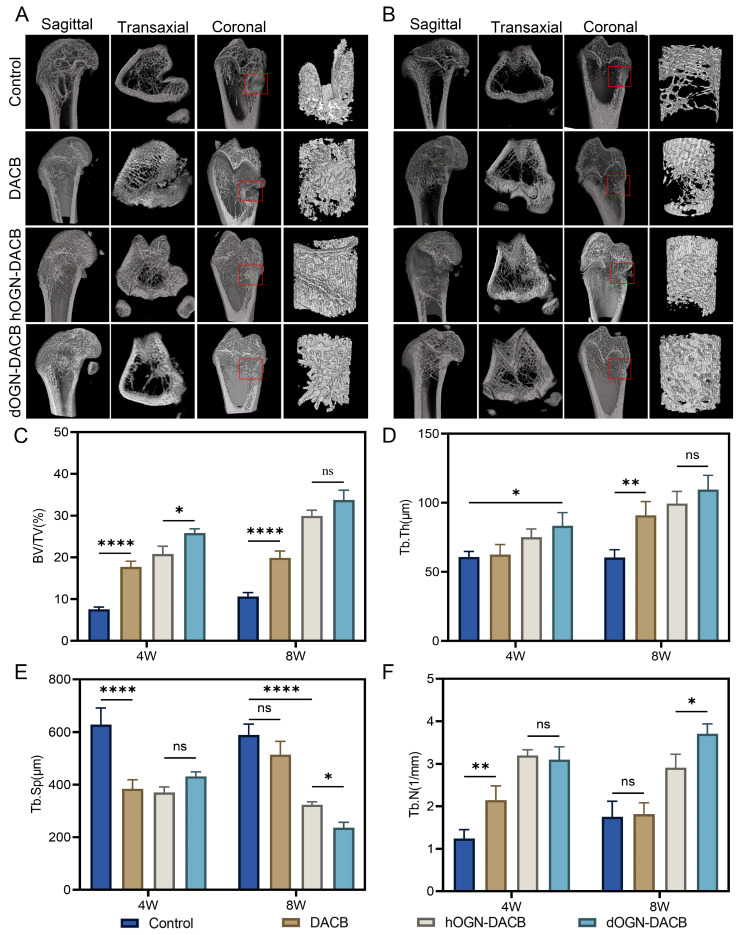
Micro-CT analysis of bone tissue microarchitecture. Representative 2D and 3D reconstructed micro-CT images of trabecular bone in the rat femur at 4 weeks (**A**) and 8 weeks (**B**). The red box indicates the defect site. (**C**–**F**) Quantitative analysis of bone morphometric parameters, including bone volume/tissue volume (BV/TV), trabecular thickness (Tb.Th), trabecular separation (Tb.Sp), and trabecular number (Tb.N). ns, not significant, * *p* < 0.05, ** *p* < 0.01 and **** *p* < 0.0001.

To evaluate bone formation in vivo, a 3 mm diameter, 4 mm deep bone defect was created in the femoral condyle and different scaffolds were implanted for 4 and 8 weeks (Figure S2). New bone formation was assessed using micro-CT imaging, with quantitative evaluations including bone volume as a percentage of total tissue volume (BV/TV), trabecular thickness (Tb.Th), trabecular number (Tb.N), and trabecular separation (Tb.Sp). As shown in Figure 7A,B, significant new bone formation was observed around the defect margins in the dOGN-DACB group at both 4 and 8 weeks, compared to the control group, where no notable new bone formation was observed. As shown in Figure 7C–F, at 4 weeks, the BV/TV value in the dOGN-DACB group was 25.83 ± 1.0%, significantly higher than that of the control group (7.55 ± 0.6%) and the DACB group (17.70 ± 1.4%). After 8 weeks, the dOGN-DACB group exhibited a maximum BV/TV value of 33.74 ± 2.4%, significantly outperforming the control group (10.59 ± 1.0%) and the DACB group (22.88 ± 1.6%). Similarly, the hOGN-DACB group showed significantly higher BV/TV, Tb.Th, and Tb.N values, along with lower Tb.Sp values, compared to the other groups. 

**Figure 8:**
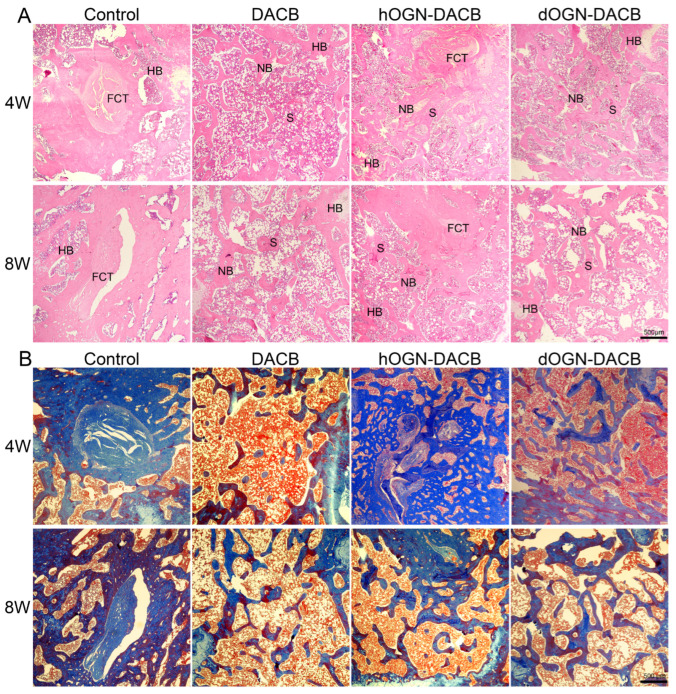
The histological analysis of the bone healing. (**A**) H&E staining and (**B**) Masson’s trichrome staining of bone defect sections at 4 and 8 weeks post-operation. FCT: fibrous connective tissue; HB: host bone; NB: new bone; S: residual material. Scale bar: 500 μm.

The new bone tissue in the defect area was evaluated histologically by H&E staining and Masson trichrome staining. As shown in Figure 8A, consistent with the micro-CT analysis at weeks 4 and 8, H&E staining in the scaffold graft group observed good tissue fusion between the implanted scaffold material and the surrounding bone tissue and new bone tissue formation in the central region of the bone defect, with no obvious inflammatory reaction or necrosis. In contrast, the defect area in the control group was mainly filled with fibrous tissue and almost no new bone formation was seen. Among them, the DACB group had sparse bone tissue in the bone margin and trabecular region at weeks 4 and 8, with limited bone regeneration. Notably, both hOGN-DACB and dOGN-DACB groups demonstrated excellent integration with host bone, exhibiting abundant mature bone tissue and bone marrow at defect margins and within scaffold pores. However, the hOGN-DACB group showed newly regenerated bone accompanied by partially formed fibrous connective tissue, whereas the dOGN-DACB group showed no fibrous tissue in H&E staining results. Additionally, Masson trichrome staining was performed to assess the deposition of collagen fibers in the newly formed bone tissue. As shown in Figure 8B, at 8 weeks, the DACB, hOGN-DACB, and dOGN-DACB groups exhibited abundant collagen fiber deposition on new bone surfaces, forming a collagen-dense and structurally compact matrix. The thickness of new bone in hOGN-DACB and dOGN-DACB groups approximated that of host bone. Combining micro-CT and histological results, dOGN-DACB restored both the morphological structure and physiological function of bone tissue. By contrast, fibrous tissue formation in hOGN-DACB during bone regeneration likely resulted from hOGN protein-mediated cellular effects. Previous reports show hOGN overexpression induces fibrosis, consistent with the fibrous tissue observed in the hOGN-DACB group [35].

**Figure 9:**
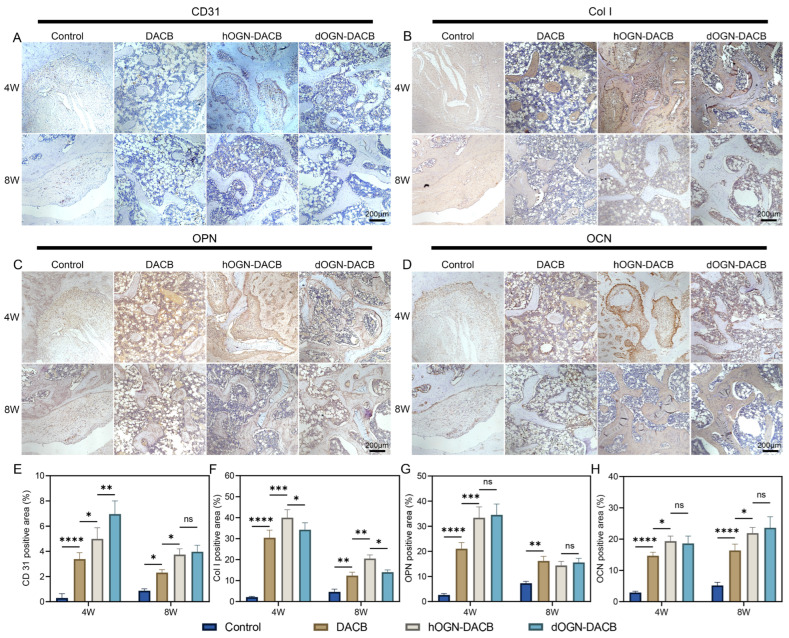
Immunohistochemical analysis of healing at 4 and 8 weeks. (**A**–**D**) IHC staining of CD31, Col I, OPN, and OCN. Scale bar: 200 μm. (**E**–**H**) Quantification of the positive area for CD31, Col I, OPN, and OCN. ns, not significant, * *p* < 0.05, ** *p* < 0.01, *** *p* < 0.001, and **** *p* < 0.0001.

To further evaluate the expression of angiogenic and bone-related proteins during bone regeneration, immunohistochemical staining of bone tissue samples was performed in this study to detect the expression levels of neovascularization markers (CD31), structural proteins (Col I), and osteoprogenitor-associated proteins (OPN and OCN) in the defect areas. Angiogenesis plays a crucial role in the early stages of bone regeneration [36,37]. As shown in Figure 9A,E, compared with the control group, CD31 expression in the DACB, hOGN-DACB, and dOGN-DACB groups peaked at week 4 and was significantly higher than that at week 8. At week 4, the dOGN-DACB group had the largest CD31-positive area, which indicated that this group had more active angiogenesis at the early stage, and the abundant blood vessel regeneration helped to provide a good microenvironmental support for the subsequent bone tissue regeneration [38]. Subsequently, CD31 expression decreased, reflecting the transition of the bone regeneration process from angiogenesis dominated in the early stage to bone remodeling dominated in the late stage [39,40].

During early bone regeneration, osteoblasts synthesize abundant Col I to form a mineralization foundation [41]. As shown in Figure 9B,F, hOGN-DACB and dOGN-DACB groups showed significantly higher Col I expression than the control at week 4, indicating stronger early osteogenic activity. Notably, hOGN-DACB’s largest positive staining area at week 4 resulted from both osteoblast-driven matrix deposition and hOGN-induced fibrous tissue collagen synthesis, as confirmed by H&E staining (Figure 8A). By week 8, Col I expression decreased in all groups, aligning with the matrix synthesis-to-mineralization transition. In contrast, dOGN-DACB showed localized Col I expression in new bone without fibrous tissue, indicating dOGN promotes collagen-mineralization coupling via precise osteogenic regulation, unlike hOGN’s fibroblast induction. This highlights dOGN’s selective osteoblast activation during bone repair.

In addition, OPN and OCN are important markers in the middle and late stages of bone formation, and their expression levels are closely related to the mineralization and remodeling of bone tissues [42]. OPN is involved in the early construction of bone matrix by regulating the alignment of Col I, promoting the resorption of immature bone and the attachment of osteoclasts in the early stage [43]. As shown in Figure 9C,G, OPN expression was significantly upregulated at week 4, and was still expressed at week 8 but with a more dispersed distribution and relatively lower concentration, reflecting that the regenerated bone tissue was close to mature bone tissue at week 8 after transplantation scaffold of DACB, while hOGN-DACB and dOGN-DACB scaffolds had nearly reached maturity. As shown in Figure 9D,F, OCN was continuously elevated from week 4 to week 8, indicating that OCN was involved in the process of bone matrix stabilization and mineral deposition, and the loading of dOGN enhanced its expression level. Finally, H&E staining of the major organs (heart, liver, spleen, lungs, and kidneys) in the experimental animals revealed no significant inflammatory infiltration or tissue edema, further confirming the good in vivo biocompatibility and safety of DACB, hOGN-DACB, and dOGN-DACB (Figure S3). 

In summary, this study confirms that dOGN significantly enhances cell proliferation and migration while exhibiting potent osteogenic differentiation-inducing activity. When loaded onto the DACB surface, dOGN’s sustained-release properties at the defect site enhance DACB bioactivity, thereby promoting bone tissue repair. During bone repair, dOGN showed stronger antifibrotic ability than hOGN. Notably, although the immunogenic mechanisms underlying dOGN-mediated bone regeneration, the pathways of vascular network formation, and the mechanistic basis for functional differences between dOGN and hOGN require further investigation through multi-dimensional in vitro and in vivo assays, existing evidence clearly establishes dOGN’s core regulatory role in promoting osteogenic differentiation. This provides critical theoretical support for subsequent molecular mechanism elucidation and functional design of bone repair biomaterials.

## 4. Conclusions

In this study, we successfully purified dOGN protein in vitro and confirmed its bioactivity by evaluating its effects on cell proliferation and migration. Subsequently, loading dOGN onto DACB further enhanced its ability to promote cell proliferation, adhesion, and osteogenic differentiation. As a bioactive scaffold material, DACB provided an optimal microenvironment for bone repair, while the incorporation of dOGN further amplified its regenerative potential. In a rat femoral condyle defect model, dOGN-DACB significantly enhanced bone tissue regeneration compared to DACB alone. These findings suggest that dOGN is a promising bioactive factor for bone tissue engineering, capable of promoting bone defect regeneration and restoring physiological function.

## Data Availability

The original contributions presented in this study are included in the article and Supplementary Material. Further inquiries can be directed to the corresponding authors.

## Supplementary Materials

The following supporting information can be downloaded at: https://www.mdpi.com/article/10.3390/biom15081124/s1, Figure S1: Cumulative release profiles of dOGN and hOGN from the corresponding dOGN-DACB and hOGN-DACB composites; Figure S2: Rat femur specimens were obtained 4 or 8 weeks after implantation of the different composites. Black circles represent defective areas. Figure S3: HE staining images of major organs when different scaffolds implanting for 8 weeks. Scare bar: 250 μm. Table S1: Primer sequences used for RT-qPCR analysis.
