# Supplementary material for: Promotion of Bone Defect Repair Using Decellularized Antler Cancellous Bone Loaded with Deer Osteoglycin
Source: Biomolecules. 2025 Aug 4;15(8):1124. doi: 10.3390/biom15081124 (PMC12383809; doi:10.3390/biom15081124)
Supplement: Supplementary file 1 [file biomolecules-15-01124-s001.zip › biomolecules-3694587-supplementary.pdf]

Supporting information

Table S1. Primer sequences used for RT-qPCR analysis.

**Osteogenic gene**	**Primer sequence (5–3′)**
OCN	Forward GCAATAAGGTAGTGAACAGACTCC
	Reverse CCATAGATGCGTTTGTAGGCGG
OPN	Forward GCTTGGCTTATGGACTGAGGTC
	Reverse CCTTAGACTCACCGCTCTTCATG
Col I	Forward TGGAGAGAGCATGACCGATG
	Reverse GAGCCCTCGCTTCCGTACT
GAPDH	Forward CATGGCCTTCCGTGTTCCTA
	Reverse GTTGAAGTCGCAGGAGACAAC

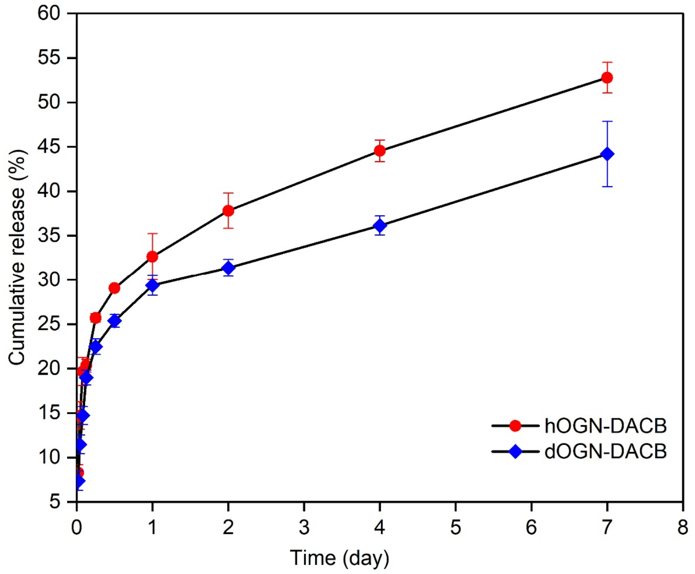

Figure S1. Cumulative release profiles of dOGN and hOGN from the corresponding dOGN-DACB and hOGN-DACB composites.

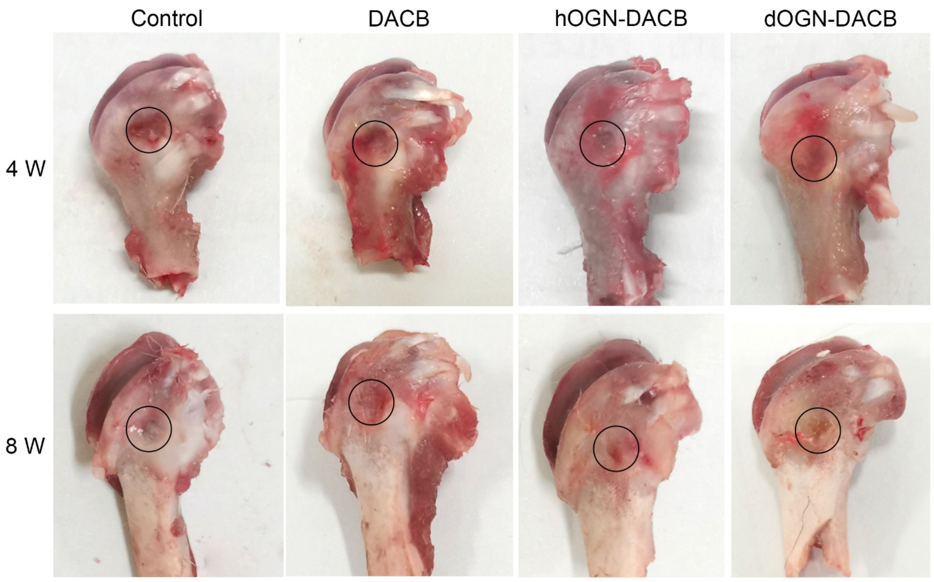

Figure S2. Rat femur specimens were obtained 4 or 8 weeks after implantation of the different composites. Black circles represent defective areas.

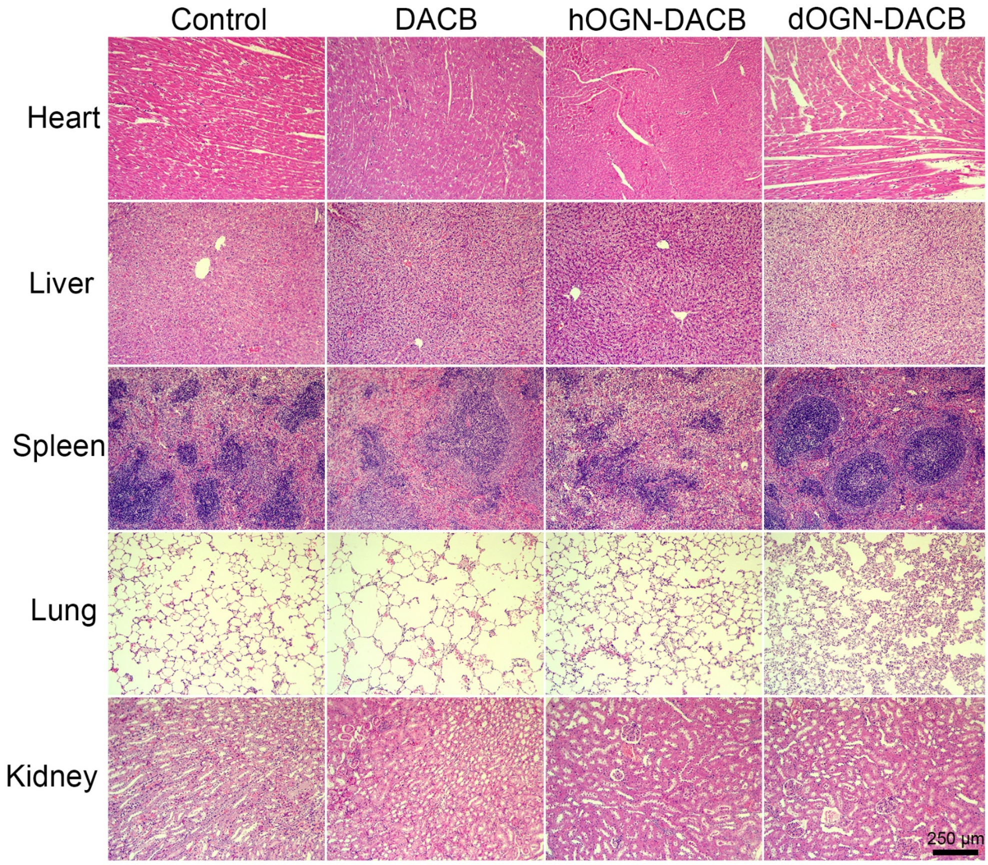

Figure S3. HE staining images of major organs when different scaffolds implanting for 8 weeks. Scare bar: 250 μm.
